# High Frequency of Blackwater Fever Among Children Presenting to Hospital With Severe Febrile Illnesses in Eastern Uganda

**DOI:** 10.1093/cid/cix003

**Published:** 2017-01-19

**Authors:** Peter Olupot-Olupot, Charles Engoru, Sophie Uyoga, Rita Muhindo, Alex Macharia, Sarah Kiguli, Robert O. Opoka, Samuel Akech, Carolyne Ndila, Richard Nyeko, George Mtove, Julius Nteziyaremye, Martin Chebet, Elizabeth C. George, Abdel G. Babiker, Diana M. Gibb, Thomas N. Williams, Kathryn Maitland

**Affiliations:** 1 Mbale Regional Referral Hospital Clinical Research Unit,; 2 Busitema University Faculty of Health Sciences, Mbale Campus; 3 Soroti Regional Referral Hospital, Soroti, Uganda; 4 Kenya Medical Research Institute–Wellcome Trust Research Programme, Kilifi; 5 Makerere College of Health Sciences, Department of Paediatrics, Kampala, and; 6 St Mary’s Hospital, Lacor, Uganda; 7 Joint Malaria Programme, Teule Hospital, Muheza, Tanzania; and; 8 Medical Research Council, Clinical Trials Unit, University College London, and; 9 Faculty of Medicine, Imperial College, London, United Kingdom

**Keywords:** African child, malaria, blackwater fever, hemoglobinuria, haemoglobinopathies

## Abstract

**Background.:**

In the Fluid Expansion as a Supportive Treatment (FEAST) trial, an unexpectedly high proportion of participants from eastern Uganda presented with blackwater fever (BWF).

**Methods.:**

We describe the prevalence and outcome of BWF among trial participants and compare the prevalence of 3 malaria-protective red blood cell polymorphisms in BWF cases vs both trial (non-BWF) and population controls.

**Results.:**

Of 3170 trial participants, 394 (12.4%) had BWF. The majority (318 [81.0%]) presented in eastern Uganda and were the subjects of further analysis. BWF cases typically presented with both clinical jaundice (254/318 [80%]) and severe anemia (hemoglobin level <5 g/dL) (238/310 [77%]). *Plasmodium falciparum* parasitemia was less frequent than in non-BWF controls, but a higher proportion were positive for *P. falciparum* histidine rich protein 2 (192/246 [78.0%]) vs 811/1154 [70.3%]; *P* = .014), suggesting recent antimalarial treatment. Overall, 282 of 318 (88.7%) received transfusions, with 94 of 282 (33.3%) and 9 of 282 (3.4%) receiving 2 or 3 transfusions, respectively. By day 28, 39 of 318 (12.3%) BWF cases and 154 of 1554 (9.9%) non-BWF controls had died (*P* = .21), and 7 of 255 (3.0%) vs 13/1212 (1%), respectively, had severe anemia (*P* = .036). We found no association with G6PD deficiency. The prevalence of both the sickle cell trait (10/218 [4.6%]) and homozygous α^+^thalassemia (8/216 [3.7%]) were significantly lower among cases than among population controls (334/2123 [15.7%] and 141/2114 [6.6%], respectively), providing further support for the role of malaria.

**Conclusions.:**

We report the emergence of BWF in eastern Uganda, a condition that, according to local investigators, was rare until the last 7 years. We speculate that this might relate to the introduction of artemisinin-based combination therapies. Further studies investigating this possibility are urgently required.

## Summary

We have noted a recent upsurge in blackwater fever among children presenting to hospitals in eastern Uganda and speculate that this may relate to a change in policy toward new artemisinin-based combination therapies for malaria.

Historically, blackwater fever (BWF), a syndrome comprising acute intravascular hemolysis, fever, and the passage of dark or red-colored urine, has been recognized most commonly as a complication of recent or concurrent *Plasmodium falciparum* malaria infection in nonimmune expatriate adults living in Africa, where it was frequently complicated by severe anemia, jaundice, and renal failure [1, 2]. Although the etiology of BWF has been the subject of considerable speculation, it has frequently been linked to prior treatment with either quinine [3] or synthetic arylamino alcohol antimalarials [4] such as mefloquine and halofantrine, or to glucose-6-phosphate dehydrogenase (G6PD) deficiency [5]. A causal association between BWF and quinine is supported by its virtual disappearance from Africa following its replacement by chloroquine [6]. BWF has generally been considered a rare complication of malaria in African children [7], although a number of case reports and case series have variously linked the condition to the use of quinine [7, 8] or to G6PD deficiency [9]. More recently, interest in BWF has been renewed following reports of a form of delayed hemolytic anemia with a more benign clinical course (postartemisinin delayed hemolysis [PADH]) that has been linked to the treatment of severe malaria with intravenous artesunate [10]. Initially described in travelers returning to Europe, PADH has also been described in case reports and small series in malaria-endemic regions of Africa [11] and Southeast Asia [10, 12].

Our recently reported multicenter trial of fluid resuscitation in African children with severe febrile illnesses and shock (Fluid Expansion as a Supportive Treatment [FEAST]) [13] included large subgroups with sepsis and malaria. Because hemoglobinuria is included in the World Health Organization classification of severe malaria a question about dark urine was also included in the FEAST case report form. Here, we describe the prevalence, clinical features, and outcome of clinically reported dark urine (potential BWF) among children recruited to the FEAST trial.

## METHODS

### Patients and Methods

The methods and outcome of the FEAST trial have been described in detail previously [13]. In brief, FEAST was a pragmatic multicenter trial of fluid resuscitation conducted among children presenting to 6 East African hospitals in Kenya (1 center), Tanzania (1 center) and Uganda (4 centers) that enrolled 3170 children (aged 2 months to 12 years) presenting to hospital with severe febrile illnesses. Children with severe malnutrition, gastroenteritis, trauma, burns, and nonmedical diagnoses were excluded.

### Baseline and Follow-up Data and Sample Analyses

A structured clinical case report form was completed at admission by study clinicians. Hemoglobinuria was defined as a clinical history of red, dark brown, or cola-colored urine. Admission blood samples were collected from recruited children, and whole blood and plasma were stored at –80°C for subsequent analysis. Plasma *P. falciparum* histidine rich protein 2 (pfHRP2), a sensitive marker of recent malaria infection, was quantified using methods described in detail previously [14]. The red blood cell polymorphisms sickle cell trait (hemoglobin [Hb] AS), sickle cell anemia (HbSS), the common African variant of α^+^thalassemia, and the B, A, and A^–^ allelic variants of G6PD were typed by polymerase chain reaction [15] from DNA extracted using proprietary methods (Qiagen DNA Blood Mini Kits, Crawley, United Kingdom). With the exception of the study intervention, management of patients in the FEAST trial largely followed national guidelines. Children were monitored throughout admission. Hemoglobin estimation was repeated at 8 and 24 hours and at 28 days, and mortality was reported at 48 hours and 28 days postrandomization.

### Population Controls

To compare the prevalence of red blood cell genetic polymorphisms among case subjects with hemoglobinuria to those in the general population, we recruited a second set of representative population controls from Mbale Regional Referral Hospital (MRRH) and Soroti Regional Referral Hospital (SRRH). Following appropriate consent, 2-mL samples of umbilical cord blood were collected from infants delivered consecutively on the maternity wards of MRRH and SRRH between 10 August 2012 and 26 February 2013, along with data on ethnic group and place of residence.

### Data Analysis

First, we described the demographic characteristics and clinical and laboratory features of trial participants by clinical site. In the 2 sites with the highest prevalence of BWF (MRRH and SRRH, eastern Uganda) we then compared data from FEAST participants with a clinical history of hemoglobinuria (BWF cases) and any participant without BWF (controls). Nonnormally distributed data were log10 transformed before analysis. Continuous variables were compared using the Student’s *t* test, categorical variables using the χ^2^ test, and proportions by logistic regression. All statistical analyses were performed using Stata software, version 11.0 (StataCorp, College Station, Texas).

The FEAST protocol was approved by the ethics committees of Imperial College, London; Makerere University and the National Council for Science and Technology in Uganda; the Kenya Medical Research Institute in Kenya; and the National Medical Research Institute in Tanzania, while ethical permission for the collection of blood and data from newborn controls was granted by the Mbale Research Ethics Committee, Uganda.

## RESULTS

### Demographic and Clinical Data Across Sites

The demographic characteristics, clinical features of severity, and mortality among FEAST trial participants, stratified by study site, are summarized in Table 1. The median age of participants (24 months [interquartile range, 13–38 months]) was similar across sites. Overall, blood films were positive for *P. falciparum* malaria parasites in 1778 of 3170 (56.0%) participants, ranging from 40.7% (Kilifi, Kenya) to 73.1% (Lacor, northern Uganda). Hemoglobinuria and clinical evidence of jaundice were reported in 394 of 3170 (12.4%) and 1014 of 3164 (32.0%) participants, respectively. The majority of cases with BWF (391 [99.2%]) presented in Uganda, with a particular focus in 2 hospitals in eastern Uganda (MRRH and SRRH), where BWF was recorded in 180 of 1240 (14.5%) and 138 of 680 (21.8%) participants, respectively while jaundice was recorded in 759 (61.2%) and 170 (27.0%) cases, respectively (Table 1).

**Table 1.** Summary of the Major Clinical and Laboratory Features of Severity Within the FEAST Study Sites

Site	Mbale, Uganda	Soroti, Uganda	Lacor, Uganda	Kampala, Uganda	Kilifi, Kenya	Teule, Tanzania
No.	1240	633	234	750	216	97
Demographic features						
Malaria endemicity	Holoendemic	Holoendemic	Hyperendemic	Hyperendemic	Mesoendemic	Mesoendemic
Male sex	698 (56.3)	340 (53.7)	125 (53.4)	394 (52.5)	102 (47.2)	46 (47.4)
Age, mo^a^, median (IQR)	25 (14–39)	23 (14–36)	27 (16–36)	21 (12–39)	26 (11–46)	19 (12–39)
Clinical features of severity						
Respiratory distress	1222/1236 (99.0)	495/630 (78.6)	125 (53.4)	514/745 (69.0)	177 (82.0)	77/96 (80.2)
Coma	241/1239 (19.5)	61/631 (9.7)	30 (12.8)	57 (7.6)	57 (26.4)	30 (31.0)
Jaundice	759 (61.2)	170/630 (27.0)	28 (12.0)	55/747 (7.4)	1 (0.5)	1 (1.0)
Dark urine	180 (14.5)	138/632 (21.8)	27 (11.5)	46/747 (6.2)	1 (0.5)	2 (2.0)
Laboratory data						
Malaria positive	699 (56.4)	388 (61.3)	171 (73.1)	381 (51.0)	88 (40.7)	51 (52.6)
Hemoglobin level, g/dL, mean (SD)	6.5 (3.1)	6.5 (31)	7.1 (2.9)	8.1 (3.3)	8.5(2.6)	7.5 (3.1)
Severe anemia^b^	639 (51.5)	340 (53.7)	96 (41.0)	197/743 (26.5)	34 (16.0)	37 (38.0)
Hypoglycemia^c^	46/1134 (4.1)	19/618 (3.1)	21/223 (9.4)	29/710 (4.1)	9/214 (4.2)	12/95 (12.6)
Lactate >5 g/dL	466/1135 (41.1)	301/627 (48.0)	90/210 (43.0)	222/727 (30.5)	55/214 (25.7)	45/95 (47.4)
BUN >20 mmol/L	131/605 (21.7)	134/432 (31.0)	51/159 (32.1)	89/634 (14.0)	25/189 (13.2)	14/90 (15.6)
Outcome						
Mortality^d^	115 (9.3)	58 (9.2)	38 (16.2)	63 (8.4)	21 (9.7)	20 (20.6)

Data are presented as No. (proportion) unless otherwise indicated. Denominators are indicated where data are missing.

Abbreviations: BUN, blood urea nitrogen; FEAST, Fluid Expansion as a Supportive Treatment; IQR, interquartile range; SD, standard deviation.

a Completed months.

b Hemoglobin level <5 g/dL.

c Glucose <2.5 mmol/L.

d At 48 hours postrandomization.

### Clinical and Laboratory Features of BWF in Eastern Uganda

The baseline characteristics of BWF cases (n = 318) and non-BWF controls (n = 1552) at MRRH and SRRH are summarized in Table 2. Compared to controls, cases were significantly older (Table 2; Supplementary Figure 1) and were more likely to be male (*P* = .05). Cases had lower median hemoglobin concentrations (3.7 vs 7.1 g/dL; *P* < .0001), had a higher prevalence of severe anemia (77.0% vs 37.4%; *P* < .0001), were more likely to be hypothermic or clinically jaundiced or to have features of impaired perfusion, and displayed greater evidence of metabolic acidosis and acute renal injury. Both mean blood urea nitrogen (BUN) and the prevalence of hyperkalemia were >2-fold higher among cases than controls. *Plasmodium falciparum* parasitemia, detected either by rapid test (Optimal IT, Diamed, Switzerland), which can remain positive for some weeks after a malaria event, or microscopy, was significantly less common in cases than controls; however, pfHRP2 positivity was significantly more common (78.0% vs 70.3% in cases and controls, respectively; *P* = .014). Moreover, among those testing positive, geometric mean concentrations of pfHRP2 were lower in cases than in controls (200 vs 613 ng/mL, respectively; *P* < .0001), suggesting a higher rate of recent antimalarial treatment in cases compared with controls. *Plasmodium falciparum* was the only species of malaria parasites detected by either microscopy or by Optimal.

**Table 2.** Clinical Characteristics and Laboratory Parameters of Subjects With or Without a Clinical History of Dark Urine in Mbale and Soroti Hospitals, Eastern Uganda

Characteristics	BWF	Non-BWF	*P* Value^a^
No.	318	1552	
Demographic features			
Male sex	192 (60.4)	844 (54.4)	.05
Age, mo, median (IQR)	36 (26–56)	22 (13–36)	<.0001
General clinical features			
Axillary temperature, °C, mean (95% CI)	37.4 (37.3–37.6)	38.2 (38.0–38.2)	<.0001
Hypothermia <36.0°C	35 (11.0)	97 (6.3)	<.0001
Clinical pallor	263 (83.0)	515/1551 (33.2)	<.0001
Clinical jaundice	254 (80.0)	673 (43.4)	<.0001
Respiratory system			
Respiratory distress	275/317 (87.0)	1439/1546 (93.1)	<.0001
Crackles (pneumonia)	38 (12.0)	359 (23.1)	<.0001
Circulatory system			
Tachycardia^b^	180 (57.0)	1085/1544 (70.3)	<.0001
Capillary refill time (≥3 sec)	170 (53.5)	348/1550 (22.5)	<.0001
Temperature gradient	224 (70.4)	968 (62.4)	.006
Weak pulse volume	92 (29.0)	361 (23.3)	.032
Decreased skin turgor (dehydration)	35 (11.0)	127 (8.2)	.103
Systolic blood pressure, mm Hg, median (IQR)	89 (82–97)	92 (85–101)	.001
Hypotension^c^	28/312 (9.0)	92/1514 (6.1)	.12
Nervous system			
Prostrate	239 (75.2)	824/1549 (53.2)	<.0001
Coma	27 (8.5)	274/1549 (17.7)	<.0001
Convulsions at admission	14 (4.4)	209/1549 (13.5)	<.0001
Hematology and biochemistry			
Hemoglobin, g/dL, median (IQR)	3.7 (2.9–4.8)	7.1 (4.3–9.7)	<.0001
Lactate, mmol/L, median (IQR)	7.9 (3.7–13.2)	3.7 (2.2–7.7)	<.0001
Severe lactatemia (≥5 mmol/L)	204/309 (66.0)	560/1450 (38.6)	<.0001
Anion gap, mean (95% CI)	20.2 (19.4–21.0)	17.2 (16.8–17.6)	<.0001
Elevated anion gap (>11.0 mEq/L)	75/172 (43.6)	153/783 (19.5)	<.0001
Glucose, mmol/L, median (IQR)	7.9 (5.9–10.2)	7.0 (5.6–8.8)	.0011
Sodium, mean (95% CI)	135.0 (134.3–135.8)	133.6 (133.2–133.9)	.0007
Potassium, mean (95% CI)	4.7 (4.5–4.8)	4.2 (4.1–4.3)	<.0001
Hyperkalemia (≥5.0 mmol/L)	32/189 (17.0)	69/836 (8.3)	<.0001
BUN, mmol/L, mean (95% CI)	33.0 (29.1–36.7)	14.2 (13.2–15.1)	<.0001
High BUN (>20 mmol/L)	123/187 (65.8)	140/847 (16.5)	<.0001
Infection			
Malaria blood slide positive	147/300 (49.0)	938/1484 (63.2)	<.0001
pfHRP2 positive	192/246 (78.0)	811/1154 (70.3)	.014
Malaria blood slide or pfHRP2 positive	192/318 (60.4)	1148/1552 (74.0)	<.0001
Geometric mean pfHRP2, ng/mL (95% CI)	200 (149–269)	613 (541–695)	<.0001
HIV antibody positive	4/258 (1.6)	37/1160 (3.2)	.022

Data are presented as No. (proportion) unless otherwise indicated.

Abbreviations: BUN, blood urea nitrogen; BWF, blackwater fever; CI, confidence interval; HIV, human immunodeficiency virus; IQR, interquartile range; pfHRP2, *Plasmodium falciparum* histidine-rich protein 2.

a *P* values reflect χ^2^ test for comparisons of proportions and Student’s *t* test for comparisons of means.

b Pulse rate of >120 beats per minute (bpm) in children aged >5 years, >140 bpm in children aged 1–4 years, and >160 bpm in children aged <1 year.

c Hypotension reflects systolic blood pressure <50 mm Hg, <60 mm Hg, and <70 mm Hg for children aged <1, 1–4, and >5 years, respectively.

### Urinalysis

For logistic reasons, urine samples were collected and tested at admission only from a subset of case (n = 100) and control (n = 366) children (Table 3). Moreover, the Multistix test (Multistix® 10 SG, Siemens) was uninterpretable (and not reportable) when urine was profoundly black or cola-colored. Both hemolyzed and nonhemolyzed red blood cells were detected more frequently in cases than controls whereas positivity for urobilinogen, a marker of intravascular hemolysis, was low among both. Ketonuria and high specific gravity were common in both groups, likely reflecting the severity of the acidosis and hypovolemia. Because urinary tract infection was rarely recorded as a final diagnosis at 48 hours, we suspect that the presence of leukocyte esterase (indicative of possible urinary tract infection) may have reflected contamination due to nonsterile sample collection techniques and/or delays in processing [13].

**Table 3.** Urine Dipstick Results in Patients With and Without a Clinical History of Blackwater Fever

Parameter	With Blackwater Fever, no./No. (%)	Without Blackwater Fever, no./No. (%)	*P* Value
Hemolyzed RBCs	51/94 (54.3)	46/314 (14.6)	<.0001
Nonhemolyzed RBCs	10/89 (11.2)	19/297 (6.4)	.3
High bilirubin	22/100 (22.0)	59/362 (16.3)	.23
High urobilinogen	8/100 (8.0)	32/358 (9.0)	.94
Ketones	57/100 (57.0)	144/364 (40)	.001
Proteinuria	65/100 (65.0)	167/366 (45.6)	.001
Nitrate	4/100 (4.0)	28/364 (7.7)	.332
Glycosuria	1/100 (1.0)	5/366 (1.4)	.551
Aciduria	69/100 (69.0)	271/359 (75.5)	.63
High specific gravity	62/100 (62.0)	187/358 (52.2)	.333
Leukocyte esterase	17/100 (17.0)	77/363 (21.2)	.5

Not all parameters were reportable where the urine was too dark to interpret.

Abbreviation: RBC, red blood cell.

### Red Blood Cell Genetic Polymorphisms

The prevalence of the red blood cell polymorphisms HbAS, HbSS, α^+^thalassemia, and G6PD deficiency among cases and among FEAST and population controls are summarized in Table 4. Of particular note, the prevalence of HbSS was higher among cases than community controls (odds ratio [OR], 4.89; 95% confidence interval [CI], 2.14–11.15; *P* < .001), providing a potential etiology in a small proportion (4.1%) of cases; however, no significant evidence was found to suggest that G6PD deficiency was a cause. Both HbAS and homozygous α^+^thalassemia were associated with protection from BWF (case-community control: OR, 0.26 [95% CI, .13–.50; *P* < .001] and 0.53 [95% CI, .25–1.11; *P* = .09], respectively), providing further evidence for the etiological role of malaria in the current case series.

**Table 4.** Inherited Red Blood Cell Disorders in Cases, Hospital Controls, and Community Controls

RBC Polymorphism	BWF Cases	Non-BWF Controls	aOR^a^ (95% CI)	*P* Value^a^	Community Controls	aOR^b^ (95% CI)	*P* Value^b^
Hemoglobin S							
AA (normal)	199/218 (91.3)	996/1137 (87.6)	Ref		1770/2123 (83.4)	Ref	
AS (sickle cell trait)	10/218 (4.6)	68/1137 (6.0)	0.67 (.33–1.33)	.25	334/2123 (15.7)	0.26 (.13–.50)	<.001
SS (sickle cell anemia)	9/218 (4.1)	70/1137 (6.4)	0.54 (.26–1.11)	.09	19/2123 (0.9)	4.89 (2.14–11.15)	<.001
α^+^Thalassemia							
Normal	126/216 (58.3)	609/1141 (53.6)	Ref		1181/2114 (55.9)	Ref	
Heterozygote	82/216 (38.0)	447/1141 (38.5)	0.89 (.65–1.21)	.48	792/2114 (37.5)	0.93 (.69–1.25)	.66
Homozygote	8/216 (3.7)	85/1141 (7.9)	0.45 (.21–.96)	.03	141/2114 (6.6)	0.53 (.25–1.11)	.09
G6PD deficiency							
Normal	189/224 (84.4)	935/1061 (88.1)	Ref		531/597 (88.9)	Ref	
Deficient^c^	35/224 (15.6)	126/1061 (11.9)	1.37 (.91–2.05)	.12	66/597 (11.1)	1.46 (.92–2.29)	.10

Data are presented as the number with each condition/total number with data (proportion).

Abbreviations: aOR, adjusted odds ratio; BWF, blackwater fever; CI, confidence interval; G6PD, glucose-6-phosphate dehydrogenase; RBC, red blood cell.

a Odds ratios and *P* values for each genotype in cases vs hospital controls estimated by logistic regression, adjusting for sex and location.

b Odds ratios and *P* values for each genotype in cases vs community controls estimated by logistic regression, adjusting for sex and location.

c Male hemizygotes and female homozygotes for the G6PD A^–^ polymorphism. Sample collection and storage was delayed at the beginning of the study, meaning that samples were not availabel for all participants.

### Follow-up and Outcome in Eastern Uganda

Mean hemoglobin level in cases remained significantly lower at 8 hours and 24 hours (Table 5). Overall, 282 of 318 cases (88.7%) received a transfusion compared with 700 of 1552 controls (45.1%) (*P* < .0001). Moreover, a higher proportion of cases than controls received a second and third transfusion (94/282 [33.3%] vs 107/700 [15.3%] and 9/282 [3.4%] vs 10/700 [1.4%], respectively; *P* < .0001). Despite a greater number of severity features in cases, 48-hour mortality was similar to that of controls. By day 28, both case fatality and the proportion with severe anemia were marginally higher in cases than controls, whereas malaria infection remained significantly lower among cases (Table 5).

**Table 5.** Hematological Indices and Outcome Over Time

Outcome Measure	With BWF (n = 318)	Without BWF (n = 1552)	*P* Value
Hemoglobin at 8 h, g/dL, mean (95% CI)	5.8 (5.6–6.0)	7.4 (7.3–7.6)	<.0001
Severe anemia^b^ at 8 h	103 (32.4)	189/1552 (12.2)	<.0001
Hemoglobin at 24 h, g/dL, mean (95% CI)	6.5 (6.2–6.7)	7.7 (7.6–7.8)	<.0001
Severe anemia^b^ at 24 h	56 (17.6)	92 (6.0)	<.0001
Hemoglobin at day 28, g/dL, mean (95% CI)	10.7 (10.4–10.9)	10.2 (10.1–10.3)	.0009
Severe anemia^b^ at day 28	7/255 (3.0)	13/1212 (1.0)	.036
Malaria blood slide positive at 28 d	35/248 (14.1)	240/1186 (20.0)	.026
Mortality at 48 h	33 (10.4)	138 (8.9)	.40
Cumulative mortality at 28 d	39 (12.3)	154 (9.9)	.21

Data are presented as No. (proportion) unless otherwise indicated. The percentages assume that all children are alive at 8 hours and 24 hours.

Abbreviations: BWF, blackwater fever; CI, confidence interval.

a Hemoglobin level <5 g.

## DISCUSSION

Historically, BWF has been considered a rare complication of malaria among children living in high-transmission settings [16–18]. More recently, however, a growing number of pediatric case series have been published from both Africa [8, 19–22] and Oceania [23]. An early report from Lagos, Nigeria [19], described 20 children who presented to hospital with hemoglobinuria that, in large part, the authors ascribed to G6PD deficiency (15/17 patients tested). Only 2 of these cases were malaria positive. In another study conducted in the same country [20], hemoglobinuria (defined by dipstick testing of urine) was described in 48 of 251 (19.1%) children presenting to hospital with severe malaria, and was commonly associated with clinical jaundice (28/48 [58.3%]). In studies conducted in the Democratic Republic of Congo (DRC) [8] and Senegal [24], evidence was found to suggest that exposure to quinine might be an important etiological factor. Finally, in a careful evaluation of children presenting with severe malaria in Papua New Guinea, O’Donnell and colleagues [23] found that although clinically apparent BWF was relatively rare (22/351 [6%]), evidence of hemolysis was found on the basis of urine testing in a high proportion of children (115/351 [33%]).

**Figure 1.**
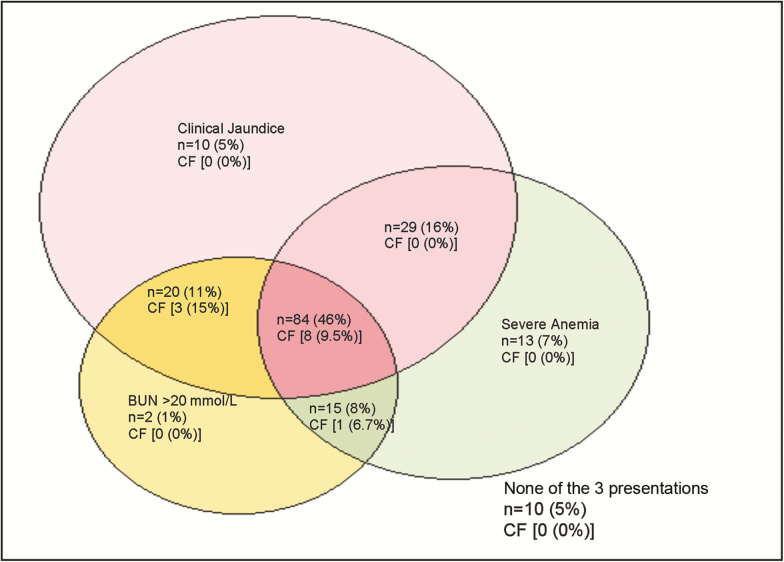
Severe anemia, jaundice, and raised blood urea nitrogen in 183 children with blackwater fever. Only 183 of 318 (56%) children had complete data on all parameters.

Abbreviations: BUN, blood urea nitrogen; CF, 48-hour case fatality.

In the current study, we observed notable geographic variation in the frequency of BWF. The majority of cases presented to 2 hospitals in eastern Uganda (MRRH and SRRH), where a history of dark urine was elicited in 14.5% and 21.8%, respectively. FEAST trial participants from these hospitals therefore form the main focus of the current report. In this series, 77.0% of BWF cases were severely anemic (hemoglobin level <5 g/dL) and in keeping with previous reports [8, 20] many (80.0%) were clinically jaundiced and showed laboratory evidence of renal impairment (BUN >20 mmol/L in 65.8%; Figure 1, Table 2). Renal impairment was frequently described in previous reports of BWF [5, 8, 25], and animal experiments have suggested that dehydration may play an important etiological role in such patients [25]. In our current series, urine testing was positive for either hemolyzed or nonhemolyzed blood in the majority of patients tested. Taken together, this constellation of symptoms, signs, and clinical investigations is consistent with acute intravascular hemolysis.

The high frequency of BWF seen in our case series from eastern Uganda contrasts with the other sites involved in the FEAST trial. Moreover, it is also at odds with observations from the an open label randomised comparison of injectable Artesunate and Quinine in patients with severe falciparum malaria in Africa (AQUAMAT) trial of severe pediatric malaria, conducted between 2005 and 2010 in 11 centers across 9 African countries, in which BWF was reported in only 4% of >5000 patients and in which only 2% presented with jaundice [26]. Of particular interest, the authors of the latter study noted that in children without a history of BWF at admission, a higher proportion developed hemoglobinuria in the artesunate arm (30/2591 [1.2%]) than in the quinine (18/2597 [0.7%]) arm of the trial (OR, 1.69; 95% CI, .94–3.05; *P* = .076), although the numbers affected were relatively few and reporting was limited to the period of hospital admission. The fact that our findings contrast with a number of previous malaria reports led us to question the cause for the high frequency of BWF seen in the current context.

In keeping with both historical and contemporary reports in children [8, 24], recent antimalarial treatment is a likely cause for BWF in our current case series. First, of the sites involved in the FEAST trial, MRRH and SRRH are situated in the areas of highest malaria transmission. Second, although a lower proportion of cases than controls were parasitemic, a higher proportion was positive for pfHRP2. Moreover, pfHRP2 concentrations were significantly lower in cases than controls, findings that together suggest a high rate of recent malaria treatment among children presenting with BWF. Finally, a role for malaria is also supported by the protective associations of HbAS and α^+^thalassaemia, which were similar in degree to those previously described for severe malaria [27]. In contrast to several previous reports [5, 19], G6PD deficiency did not provide an explanation for the high frequency of BWF in our current case series. Similarly, although some cases had HbSS, a condition that is frequently complicated by both acute and chronic hemolytic anemia and is often associated with clinical jaundice [28], this only provided a potential explanation for a small proportion (4.1%) of BWF cases in our current report.

If, in our current case series, the occurrence of BWF is linked to malaria, it raises questions about why, in general, BWF has not been recognized as a major health problem in African children previously. Local investigators have indicated that childhood BWF was rare at their hospitals until relatively recently, only having grown in importance during the last 7 years. Moreover, they report that some children are recurrently admitted with the triad of dark urine, anemia, and clinical jaundice and require frequent blood transfusions (P. O.-O. and C. E., personal communication). One possibility is a recent change in the local parasite population toward strains with a greater propensity to cause BWF, a hypothesis advanced by others previously [5, 25, 29]. Of note, however, cases with BWF in our current series were significantly older than controls presenting without BWF (Supplementary Figure 1), an observation consistent with the hypothesis, previously proposed [24], that BWF in children might result from recurrent historic exposure to antimalarial drugs. In a series of 378 children with severe malaria in Kinshasa, DRC, a city with holoendemic malaria, 25% of cases had BWF, of which 79% reported recent treatment with oral quinine and 6% had been treated with artemisinin-based combination therapies (ACTs) [22]. The authors suggested that the limited availability and higher cost of ACTs meant that quinine was being used increasingly as first-line treatment in uncomplicated malaria cases [30]. In Uganda, ACTs were introduced as first-line treatment in 2006; the routine use of quinine has since become uncommon (P. O.-O., personal communication). We suggest, therefore, that ACTs are the agents most likely to have been used in the prior treatment of BWF patients in the current case series.

Of particular interest in this regard, a growing number of recent case reports from Europe have described hemolysis following intravenous therapy with artemisinin (summarized in [10]). In contrast to most historical reports of BWF, where hemolysis and hemoglobinuria occur soon after treatment, patients in these case series developed hemolysis 14–31 days following intravenous artesunate and showed signs of persistent hemolytic activity for up to 6 weeks. Despite extensive investigations, no definitive cause for hemolysis has been identified [10]. This syndrome, labeled postartesunate noninfectious delayed hemolysis and anemia (PANDHA), is reported to occur in up to 25% of artesunate-treated travelers [10, 31]. Further elucidation of the mechanism of PANDHA indicates that it may be due to delayed hemolysis of previously infected red blood cells that have been damaged by artesunate-induced parasite removal by the spleen. In a case series of 60 nontransfused patients, 13 (22%) had delayed hemolysis [32], which was maximal after day 8 and greatest in those with the highest admission parasitemias, a distinct variant from the usual intravascular hemolysis associated with the acute phase of malaria. With respect to our current study, injectable artesunate was not available in the community during the period of this study, so if linked to antimalarial treatments, our findings suggest that a broader spectrum of arteminsin compounds may result in PANDHA. Worldwide data on this possibility remain scarce [21, 31].

Our study has a number of limitations of which perhaps the most important was the fact that case ascertainment was based on a clinical history of dark urine collected from a parent. We did not inquire about recent antimalarial treatments, and our study would have benefited from additional real-time laboratory investigations aimed at investigating the etiology more definitively. Urine testing was not possible in every child recruited and was only hemoglobin-positive in 65% of readable samples. This observation might suggest that hemoglobinuria was not present at the time of testing, but might also be attributable to the paroxysmal nature of this condition (P. O.-O., personal communication). Nevertheless, the hematological picture (median hemoglobin level 3.7 g/dL) is indicative of hemolysis, as is the high frequency of transfusion (88%) and repeat transfusion (36% of BWF cases receiving multiple transfusions). Despite this, severe anemia was significantly more common in cases than controls at 8 and 24 hours and at 28 days postadmission. This condition therefore is associated with a greater risk of severe and life-threatening morbidity and a marginal increase in the risk of longer-term mortality. Although the specific pathogenesis of PANDHA remains unclear, detailed investigation of patients in Europe did not implicate either drug-dependent or drug-independent hemolysis, although, to the best of our knowledge, a complement-mediated mechanism has yet to be investigated. A further limitation of the study is that we lack data on the prevalence of infections other than malaria. At the time the study was conducted, facilities for bacteriological culture were limited at the Mbale and Soroti sites, and too few samples were cultured to yield meaningful results.

## CONCLUSIONS

Historically, BWF has not been considered a common complication of malaria among children living in areas of high malaria transmission. Nevertheless, in the current report we have described a high frequency of BWF among participants of the FEAST trial. The majority of cases presented to 2 hospitals in eastern Uganda that are both situated within an area of very high malaria transmission. BWF has a high hidden burden of morbidity and mortality, with 90% requiring transfusions and >30% requiring multiple transfusions. Moreover, 28-day mortality was 12%, and 3% of survivors remained severely anemic at this time point. We conclude that malaria and its treatment are important factors in the etiology of BWF in the study population and that this may be linked to the recent switch toward orally administered ACTs as first-line treatment for malaria within the region. Further studies investigating the incidence and etiology of childhood BWF in the context of ACTs are indicated.

## Supplementary Data

Supplementary materials are available at *Clinical Infectious Diseases* online. Consisting of data provided by the authors to benefit the reader, the posted materials are not copyedited and are the sole responsibility of the authors, so questions or comments should be addressed to the corresponding author.

## Supplementary Material

Supplement_Figure

## Notes

***Acknowledgments*.** The study group thanks the children and families who participated in the trial and the cord blood collection in eastern Uganda. We also thank Dr Charlie Woodrow, Mahidol Oxford Research Unit, Bangkok, for his assistance in the standardization of our HRP2 assay results against their reference dataset. This article is published with permission or the Director of the Kenya Medical Research Institute.

***Disclaimer*.** The funders had no role in study design, data collection and analysis, decision to publish, or preparation of the manuscript.

***Financial support*.** The FEAST study was funded by the Medical Research Council, United Kingdom (grant number G0801439, provided through the Medical Research Council Department of International Development concordat). Baxter Healthcare Sciences donated the resuscitation fluids (5% albumin and 0.9% saline). T. N. W. is funded through a Senior Research Fellowship from the Wellcome Trust (award number 091758).

***Potential conflicts of interest*.** All authors: No potential conflicts. All authors have submitted the ICMJE Form for Disclosure of Potential Conflicts of Interest. Conflicts that the editors consider relevant to the content of the manuscript have been disclosed.
