# Supplementary figures and images for: High Frequency of Blackwater Fever Among Children Presenting to Hospital With Severe Febrile Illnesses in Eastern Uganda
Source: Clin Infect Dis. 2017 Jan 19;64(7):939–46. doi: 10.1093/cid/cix003 (PMC5848229; doi:10.1093/cid/cix003)

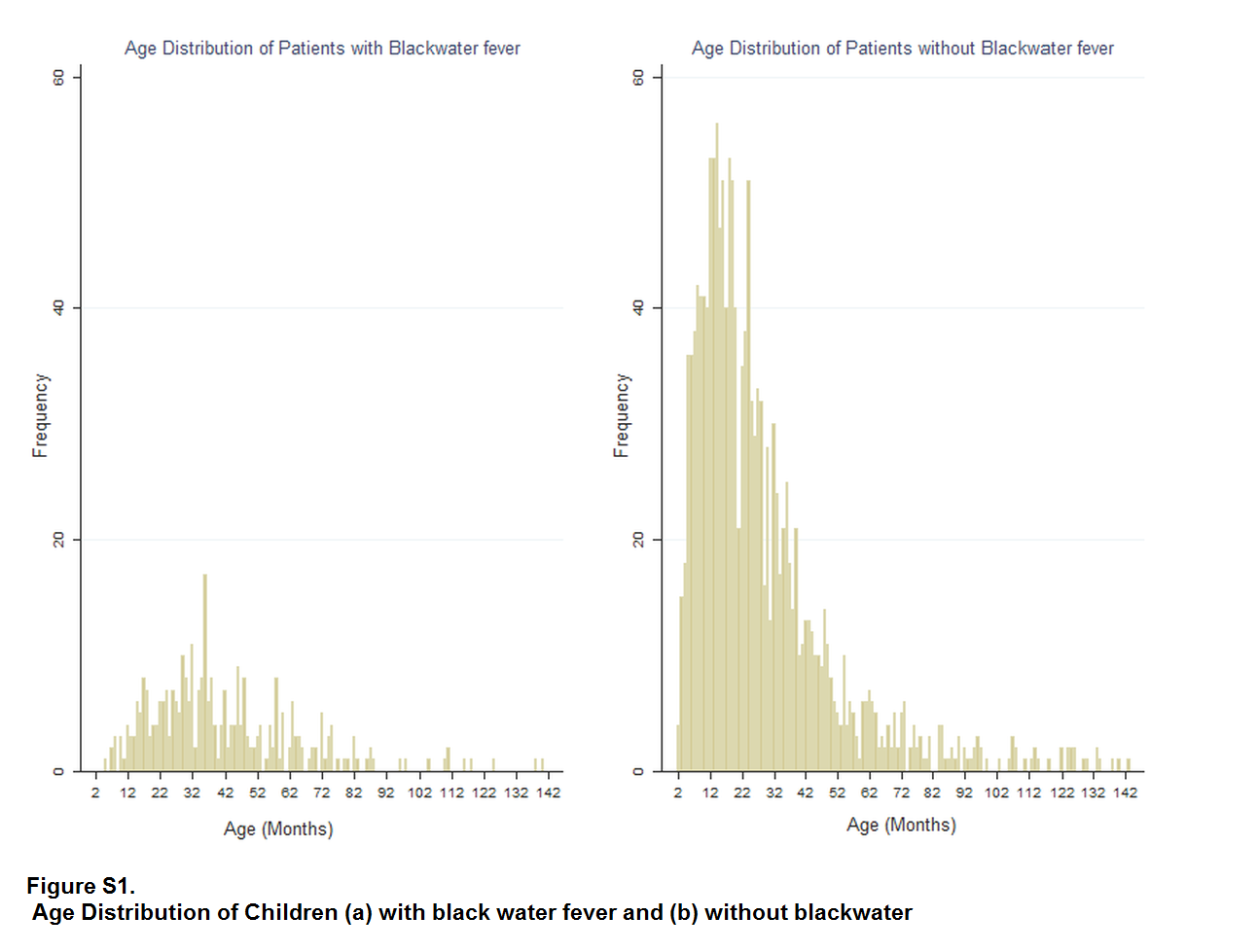

Supplement: Supplement_Figure [file cix003_supp1_supplement_figure.png]
